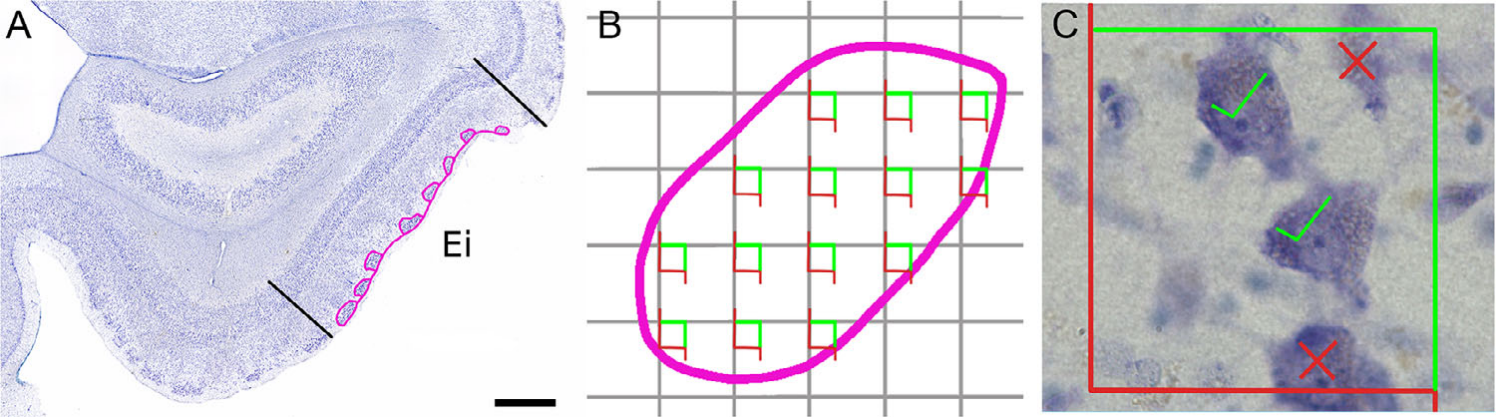# Total neuron counts in vulnerable and resilient entorhinal subregions

**DOI:** 10.1002/alz.089516

**Published:** 2025-01-03

**Authors:** Emma W Rosenblum, Jean Augustinack

**Affiliations:** ^1^ Martinos Center for Biomedical Imaging, Massachusetts General Hospital, Boston, MA USA; ^2^ Martinos Center for Biomedical Imaging, Massachusetts General Hospital, Harvard Medical School, Charlestown, MA USA; ^3^ Harvard Medical School, Charlestown, MA USA

## Abstract

**Background:**

Neuron loss exceeds tau tangle formation (Gomez‐Isla et al. 1997) and p‐tau vulnerability differs by layer and subfield in entorhinal cortex (Llamas‐Rodriguez et al., 2022). Some subfields succumb to tau early and other subfields later in Alzheimer’s disease. Investigating neuron loss by subfield is pivotal for early diagnosis.

**Method:**

We applied the optical fractionator to count neurons (West, 1991) using Stereoinvestigator (MicroBrightField Inc) in layer II subfields, ER, EI, and ECs, in n = 14 postmortem samples. The samples were preclinical controls, including 6 females, 8 males. A sampling grid (450 µm x 450 µm) with 75 µm x 75 µm counting frame was randomly overlaid. The neurons were counted using a 100x objective (Figure 1). The disector height was 10 µm; with guard zones 3 µm each. Counting inclusion criteria were 1) nucleolus in focus, 2) within inclusion lines, 3) did not touch exclusion lines. The tissue thickness was collected at each counting site (average 20.4 µm). The average coefficient of error was 0.08.

**Result:**

Total neuron counts in EI was 172,429, with ER at 133,161 and EC lower at 103,493 (averaged across samples). Total neuron counts for EI were significantly different than ECs (one‐way ANOVA, p = 0.0033, Tukey’s multiple comparison test, p = 0.0023). Total neuron number from summed subfields did not correlate with age (r = ‐0.2816). Total neuron count for males and females differed but difference was not significant (t‐test, p = 0.5669). No significant differences between total neuron number and the preclinical stages (control, Braak stage I and II samples, averaged) (ANOVA, p = 0.8732).

**Conclusion:**

These findings establish a baseline for total neuron number in specific entorhinal subregions of vulnerability (e.g., ECs) and resilience (e.g., ER, EI) at preclinical stage. These data support a previous report that total neuron number in frontal and temporal cortices does not decrease with age (Freeman et al.,2008). Related studies have applied the NvVref method (Gomez‐Isla et al. 1996, Gomez‐Isla et al. 1997, Freeman et al. 2008), but with varying tau pathology at this stage, optical fractionator is the ideal approach. The total neuron number was consistent with age, however, individual variability in the sample set was evident.